# Erratum and republication – S2k Guideline: Hygienic requirements for patient beds, bed linen, bed accessories and personal protection when handling beds

**DOI:** 10.3205/dgkh000657

**Published:** 2026-06-18

**Authors:** Axel Kramer, Julia Seifert, Bernd Gruber, Marianne Abele-Horn, Mardjan Arvand, Alexander Blacky, Michael Buerke, Iris Chaberny, Maria Deja, Steffen Engelhart, Dieter Eschberger, Anja Gerhardts, Achim Hedtmann, Julia Heider, Christian Jäkel, Peter Kalbe, Horst Luckhaupt, Wolfgang Müller, Alexander Novotny, Cihan Papan, Hansjürgen Piechota, Frank-Albert Pitten, Veronika Reinecke, Simone Scheithauer, Dieter Schilling, Walter Schulz-Schaeffer, Ulrich Sunderdiek

**Affiliations:** 1 German Society for General and Hospital Hygiene, Berlin, Germany; 2 German Trauma Society, Berlin, Germany; 3 German Nursing Council, Berlin, Germany; 4 Paul Ehrlich Society for Infection Therapy, Munich, Germany; 5 Robert Koch Institute, Department Infectious Diseases, Unit Hospital Hygiene, Infection Prevention and Control, Berlin, Germany; 6 Austrian Society for Hospital Hygiene, Vienna, Austria; 7 German Cardiac Society, Düsseldorf, Germany; 8 German Society for Hygiene and Microbiology, Münster, Germany; 9 German Society of Anaesthesiology and Intensive Care Medicine, Munich, Germany; 10 Society of Hygiene, Environmental and Public Health Sciences, Müllheim, Germany; 11 Vienna Regional Office of the Austrian Workers’ Compensation Insurance, Vienna, Austria; 12 Hohenstein Institute for Textile Innovation gGmbH (HIT), Bönningheim, Germany; 13 Professional Association of Orthopaedic and Trauma Specialists (BVOU), German Society for Orthopaedics and Trauma, Berlin, Germany; 14 German Society for Oral, Maxillofacial and Facial Surgery, Hofheim am Taunus, Germany; 15 Dr. Jäkel, Medical Law, Pharmaceuticals Law, Medical Devices Law, Luebben, Germany; 16 Professional Association of German Surgery, Berlin, Germany; 17 German Society of Oto-Rhino-Laryngology, Head and Neck Surgery, Bonn, Germany; 18 Former Head of the Office of the Association of the Scientific Medical Societies in Germany; 19 German Society for Surgery, Berlin, Germany; 20 German Society for Pediatric Infectious Diseases, Berlin, Germany; 21 German Society for Urology, Düsseldorf, Germany; 22 German-speaking Interest Group of Experts for Infection Prevention and Consultants for Hospital Hygiene, Zurich, Switzerland; 23 Department of Infection Control and Infectious Diseases, University Medical Center Göttingen (UMG), Georg-August University Göttingen, Göttingen, Germany; 24 German Society for Digestive and Metabolic Diseases, Berlin, Germany; 25 Department of Neuropathology, Medical Faculty of the Saarland University, Homburg/Saar, Germany; 26 German X-ray Society and German Society for Interventional Radiology and Minimally Invasive Therapy, Berlin, Germany

 Erratum for: Kramer A, Seifert J, Gruber B, Abele-Horn M, Arvand M, Blacky A, Buerke M, Chaberny I, Deja M, Engelhart S, Eschberger D, Gerhardts A, Hedtmann A, Heider J, Jäkel C, Kalbe P, Luckhaupt H, Müller W, Novotny A, Papan C, Piechota H, Pitten FA, Reinecke V, Scheithauer S, Schilling D, Schulz-Schaeffer W, Sunderdiek U. S2k Guideline: Hygienic requirements for patient beds, bed linen, bed accessories and personal protection when handling beds. GMS Hyg Infect Control. 2025;20:Doc20. DOI: 10.3205/dgkh000549

## Notification

This erratum notice is published to inform readers that the affiliation information for the author Scheithauer S in the article 

Kramer A, Seifert J, Gruber B, Abele-Horn M, Arvand M, Blacky A, Buerke M, Chaberny I, Deja M, Engelhart S, Eschberger D, Gerhardts A, Hedtmann A, Heider J, Jäkel C, Kalbe P, Luckhaupt H, Müller W, Novotny A, Papan C, Piechota H, Pitten FA, Reinecke V, Scheithauer S, Schilling D, Schulz-Schaeffer W, Sunderdiek U. S2k Guideline: Hygienic requirements for patient beds, bed linen, bed accessories and personal protection when handling beds. GMS Hyg Infect Control. 2025;20:Doc20. 

DOI: https://dx.doi.org/10.3205/dgkh000549

was incomplete in the original publication.

The corrected version of the article has been updated to reflect this correction:

Kramer A, Seifert J, Gruber B, Abele-Horn M, Arvand M, Blacky A, Buerke M, Chaberny I, Deja M, Engelhart S, Eschberger D, Gerhardts A, Hedtmann A, Heider J, Jäkel C, Kalbe P, Luckhaupt H, Müller W, Novotny A, Papan C, Piechota H, Pitten FA, Reinecke V, Scheithauer S, Schilling D, Schulz-Schaeffer W, Sunderdiek U. S2k Guideline: Hygienic requirements for patient beds, bed linen, bed accessories and personal protection when handling beds. GMS Hyg Infect Control. 2026;21:Doc47. 

DOI: https://dx.doi.org/10.3205/dgkh000656

The publisher regrets this error and apologizes for any inconvenience caused.

## Competing interests

The authors declare that they have no competing interests.

