# Supplementary material for: S2k Guideline: Hygienic requirements for patient beds, bed linen, bed accessories and personal protection when handling beds
Source: GMS Hyg Infect Control. 2025 May 16;20:Doc20. doi: 10.3205/dgkh000549 (PMC12172067; doi:10.3205/dgkh000549)
Supplement: Guideline Report [file HIC-20-20-s-001.pdf]

Guideline Report

What is new?

In the further development of the S1 guideline “Hygienic reprocessing of patient beds” from 2016, new findings on the role of the patient bed in the epidemiology of nosocomial infections, the reprocessing of patient beds as well as their accessories, were addressed, and the database was significantly expanded. The following new aspects were added:

1. Requirements on hospital beds to ensure restful sleep as a precondition for recovery
2. staff protection when handling beds
3. requirements for hospital laundries

Scope and purpose

The guideline addresses the prevention of nosocomial infections associated with the patient's bed in the context of medical and nursing care. It also considers the protection of staff during bed preparation.

Reasons for selecting the guideline topic

Protecting patients from nosocomial infections associated with the patient's bedside is both ethically necessary and economically relevant. This concerns both the prevention of individual infections and the prevention of outbreaks of nosocomial infections with serious illnesses and even fatal outcomes.

- **Goal orientation of the guideline**
  Transparency of the currently available evidence

- **Patient target group**
  Bedridden patients

- **User target group/addressees**
  The guideline is aimed at nursing staff and (specialist) physicians involved in in-patient care of patients as well as staff involved in the preparation of patient beds.

Guideline development process

This is the third fundamental update of an S2k guideline (first version dated 02/1999), which means it is a consensus-based guideline with a representative panel.

The guideline was developed by the Section “Hospital and Practice Hygiene of the German Society of General and Hospital Hygiene”. The participating professional societies were involved in the guideline development.

The method of the S2k guideline (consensus-based + interdisciplinary matching) is based on the recommendations of the Association of Scientific Medical Societies (AWMF). To formulate the recommendations, a systematic literature review (until January 2024) with evaluation of the sources was conduted in interdisciplinary dialogue between the participating mandate holders of the professional societies and associations.
The recommendations were graduated according to the following three-level scheme:

Description	Expression mode	Symbol
Strong recommendation	shall/shall not	⇑⇑/⇓⇓
Recommendation	should/should not	⇑/⇓
Recommendation open	can be considered/waived	⇔

Attachment to: Kramer A, Seifert J, Gruber B, Abele-Horn M, Arvand M, Blacky A, Buerke M, Chaberny I, Deja M, Engelhart S, Eschberger D, Gerhardts A, Hedtmann A, Heider J, Jäkel C, Kalbe P, Luckhaupt H, Müller W, Novotny A, Papan C, Piechota H, Pitten FA, Reinecke V, Scheithauer S, Schilling D, Schulz-Schaeffer W, Sunderdiek U. S2k Guideline: Hygienic requirements for patient beds, bed linen, bed accessories and personal protection when handling beds. GMS Hyg Infect Control. 2025;20:Doc20.
DOI: 10.3205/dgkh000549

Development of the guideline

The editorial committee prepared the draft of the updated guideline based on the literature and coordinated it with all mandate holders in attendance. Subsequently, the draft of the guideline was made available to the members of the consensus group (all mandate holders) for critical review and comment within the framework of a Delphi process with written, independent expert consultation. The comments of the members of the consensus group were integrated into a new version, submitted to the members of the editorial/consensus group for another critical review and finalized at the consensus conference.

Declaration of interests and handling of conflicts of interest

All participants in the guideline submitted their declarations of interest (AWMF table for declarations of interest in guideline projects) to the coordinator. In the table form, the participants were asked to indicate whether and, if so, which thematic reference to the guideline/guideline topic exists for the interests presented.

The following evaluation criteria were used:

- Paid expert/consultant activities for industrial companies
- Participation in a scientific advisory/advisory board: paid activity for industrial companies
- Lectures: paid by industry
- Authorship or co-authorship: only if industry controlled
- Research projects/conduction of clinical studies: directly or partly financed by industrial companies
- Ownership interests (patents, shareholdings) with guideline relevance
- Indirect interests with relevance.

Based on the table, possible conflicts of interest were discussed, and management decisions were made. For this purpose, the information was reviewed regarding an existing thematic reference, thematic relevance, type and intensity of the relationship. In the sense of active conflict of interest management, it should be decided, depending on the content of the declarations of interest of the members of the editorial committee, whether the vote of a mandate holder should be excluded from the consensus determination procedure for individual recommendations due to possible conflicts of interest. Possible conflicts of interest were classified as low/moderate/high. Low conflicts of interest due to fees for lectures (financed by relevant industry) lead to a limitation of the AG management function, but have no consequences for the vote. Possible moderate conflicts of interest were seen especially in the case of memberships in advisory boards and lecturing activities with fees from the industry, if the content was about hand hygiene issues (consequence: no vote). A high conflict of interest would have existed in the case of holding patents in connection with hand hygiene or activities predominantly for the industry (consequence: no vote and no discussion on the topic).

The relevance of activities or interests for a possible conflict of interest was assessed individually. The assessment was made and unanimous confirmation was given by the editorial committee at its meeting on September 9, 2024. The handling of interests was subsequently assessed and confirmed again jointly by all participants at the consensus conference, also on September 9, 2024. Ultimately, a moderate conflict of interest was identified in four cases. Insofar as recommendations were voted on for which the moderate conflict of interest was relevant, these four mandate holders were not considered in the consensus determination procedure.

Attachment to: Kramer A, Seifert J, Gruber B, Abele-Horn M, Arvand M, Blacky A, Buerke M, Chaberny I, Deja M, Engelhart S, Eschberger D, Gerhardts A, Hedtmann A, Heider J, Jäkel C, Kalbe P, Luckhaupt H, Müller W, Novotny A, Papan C, Piechota H, Pitten FA, Reinecke V, Scheithauer S, Schilling D, Schulz-Schaeffer W, Sunderdiek U. S2k Guideline: Hygienic requirements for patient beds, bed linen, bed accessories and personal protection when handling beds. GMS Hyg Infect Control. 2025;20:Doc20.
DOI: 10.3205/dgkh000549

Consensus finding

For consensus finding, the finalized guideline with all recommendations was provided by the editorial committee. The formal consensus process was conducted for all guideline recommendations at the consensus conference on 9/9/2024.

The following steps were followed in the consensus conference: NIH (National Institutes of Health)-type consensus conference (the recommendations were voted on under neutral moderation by Dr. rer. Biol. Hum. Cathleen Muche-Borowskias, MPH, as follows): Presentation of the recommendations to be voted on in the plenary session by the working group, opportunity for questions and submission of justified amendments, voting on the recommendations and amendments. If necessary: discussion, development of alternative proposals and final vote with following consens grading:

Strong consent	consent of >95% of the participants
Consent	consent of >75–95% of the participants
Majority consent	consent of >50–75% of the participants
No majority consent	consent of <50% of the participants

At the consensus conference on Sept. 9, 2024 (28 societies represented), approval was granted by the mandate holders for all recommendations of the guideline for all societies represented. Without exception, all recommendations were approved with “strong consensus”.
